# The fragile core of care: reframing the well-being of health professionals as critical infrastructure

**DOI:** 10.1093/haschl/qxaf123

**Published:** 2025-06-14

**Authors:** Md Doulotuzzaman Xames

**Affiliations:** Grado Department of Industrial and Systems Engineering, Virginia Tech, Blacksburg, VA 24061, United States

**Keywords:** health professionals’ well-being, healthcare system resilience, burnout, health systems design, digital twins, systems engineering, structural reform, moral injury, workforce attrition, critical infrastructure

## Abstract

While global health policy often centers patient outcomes, a dangerous oversight persists: the neglect of healthcare professionals’ well-being as foundational to system effectiveness. Burnout, attrition, and moral injury are mounting across countries, yet health systems continue to treat their workforce sustainability as peripheral. Drawing on evidence from workforce trends, burnout statistics, and systems engineering, this commentary argues that healthcare professionals’ well-being must be reframed as critical infrastructure. The piece contends that sustainability in healthcare depends on policy architectures that embed protections for health professionals, including structural supports like real-time workload monitoring, enforceable staffing ratios, and integrated mental health services. The neglect of health professionals’ well-being is not just a human resource challenge—it is a design flaw that compromises the viability of care delivery itself. Without urgent recalibration, health systems risk brittleness, inequity, and collapse under surging demands. This commentary urges a paradigm shift in how we conceptualize, design, and govern health systems, beginning with the foundational recognition that care cannot be sustainable if its health professionals are not.

## Introduction

Global health discourse has long prioritized patient outcomes, access, and equity. While ethically imperative, this patient-centric focus often obscures a structural truth: Healthcare systems depend not only on medicines, technologies, or funding, but on the labor, cognition, and emotional endurance of those who deliver care. Health professionals—including doctors, nurses, therapists, and other care team members—are not peripheral enablers of service delivery; they are its core infrastructure. Their sustained well-being is foundational to the resilience and sustainability of health systems. Yet, despite mounting evidence of burnout, moral injury, and workforce attrition—exacerbated, but not caused by, COVID-19—policy frameworks still treat health professionals’ well-being as ancillary. This is not a minor oversight; it is a systemic design failure.

WHO projected a global shortfall of 11 million health workers by 2030, disproportionately affecting low- and middle-income countries.^1^ Despite recent expansions, the NHS continues to grapple with severe staffing shortages, with approximately 111 000 posts unfilled as of 2024.^2^ While demand has surged, recruitment and retention efforts have failed to keep pace, impacting not only doctors and nurses but also physiotherapists and occupational therapists. The top reasons cited for leaving the NHS are the pursuit of better work-life balance and personal health concerns, factors that have tripled since 2013-2014.^2^ This exodus reflects a deeper structural malaise, not merely a temporary workforce issue. Globally, similar patterns prevail. In the United States, physician burnout peaked at 62.8% in late 2021.^3^ A recent survey shows that 24% of healthcare workers plan to quit their jobs within a year.^4^ Nearly 40% of US physicians plan to reduce clinical hours within a year, over twice the rate previously reported.^5^ By 2037, the United States is expected to face a shortage of approximately 187 130 physicians across all medical specialties.^6^ Female physicians face a 53% higher suicide risk compared to women in other professions,^7^ and nurses also show significantly elevated suicide rates.^8^ Meanwhile, under-resourced healthcare professionals endure unsafe staffing ratios, political volatility, and the moral distress of failing to provide standard care. These are not isolated signals—they are symptoms of a policy architecture that devalues the very agents upon whom care delivery depends.

This reality became stark during COVID-19, which exposed the fragility of healthcare systems. We applauded frontline workers, hung signs, and called them heroes. But as the crisis receded, so did the urgency to address their structural vulnerability. Many returned to the same unsustainable workloads and under-resourced environments that had pushed them to the brink. The inability of health systems to recalibrate in the face of crises underscores the fragility of the infrastructure upon which they rely. This is not simply a matter of increasing workforce numbers; it is about reshaping the system to treat health professionals’ well-being as an integral component of healthcare system resilience.

## Reframing health professionals’ well-being as critical infrastructure

As a systems engineer studying clinician burnout, I’ve spent the past 3 years immersed in the interplay between human-in-the-loop and healthcare infrastructure. The deeper I went, the more I realized the issue wasn’t just under-recognized—it was systematically overlooked. When I began my doctoral research in 2022 on using digital twins to monitor and manage clinician workload in real time,^9^ I assumed that I was building on an existing body of work. Digital twin applications were gaining traction in personalized medicine, diagnostics, and even surgical planning. But when I searched for studies applying digital twins to clinicians themselves, I found none. That absence speaks volumes. Even in innovation-driven spaces, the human side of healthcare remains underprioritized.

This research gap mirrors the broader neglect of healthcare professionals’ sustainability. If we aren’t designing technologies to support those who deliver care, who are we designing those technologies for? And if research perpetuates this oversight, how can policy or practice be expected to do better? For health systems to truly be resilient, they must account for the full spectrum of infrastructure, including the emotional, cognitive, and physical labor of their health professionals. Technologies like digital twins, which have revolutionized industries from manufacturing to urban planning, can offer a model for real-time monitoring and decision-making that would allow for continuous adjustments to clinician workloads, directly addressing key factors contributing to well-being and burnout.

Moreover, the failure to protect the health workforce reverberates throughout the system. It leads to cognitive overload, impaired clinical judgment, and rising rates of preventable harm—a direct contradiction to the patient safety goals many systems espouse. Burnout is not just an emotional state; it erodes decision quality, increases diagnostic delays, and heightens the risk of adverse events.^10^ This translates into measurable losses in system efficiency, quality, and trust.^11^ Yet, most resilience frameworks in healthcare continue to emphasize adaptive capacity at the organizational level without adequately accounting for human sustainability at the individual level. This misalignment creates a paradox: Systems are labeled “resilient” even as their core actors burn out.

## Urgent need for policy overhaul

We must urgently shift the narrative. Current interventions—wellness seminars, mindfulness workshops, crisis hotlines—are often palliative and individualized.^12^ They frame burnout as a personal failing rather than a system-level hazard.^13,14^ This reflects a deeper epistemic problem: Healthcare professionals are treated like expendable labor inputs, not critical infrastructure. We would not expect overburdened bridges to withstand surging loads without maintenance. Nor would we run power grids at peak capacity indefinitely without redundancies. Why tolerate a crumbling human foundation in healthcare?

Reframing health professionals’ well-being as critical infrastructure is not rhetorical—it's a call for structural redesign. This shift demands more than goodwill and symbolic recognition; it requires hardwired protections rooted in systems thinking, systems engineering, human factors, and resilience theory.^15^ It means investing in real-time workload monitoring tools like clinician digital twins. It means mandating minimum staffing ratios and rest periods, conducting staffing equity audits, and embedding accessible mental health services within health systems—not as fringe benefits, but enforceable standards. Accreditation bodies, reimbursement models, and performance metrics must evolve to reflect health professionals’ sustainability as a core outcome.

This reconceptualization also demands accountability mechanisms. Health systems must be evaluated on their ability to retain, protect, and support their workforce. Metrics like burnout rates, turnover, and psychological safety should be integrated into national health dashboards. However, such metrics must be carefully structured to avoid perverse incentives or erosion of psychological safety. Public reporting of well-being data may unintentionally undermine honest feedback and organizational learning by fostering reputational risk aversion rather than genuine improvement.^16^ To be effective, accountability should prioritize internal transparency, benchmarked assessments, and alignment with accreditation structures rather than punitive public rankings. Strategic workforce planning should move beyond headcounts and include assessments of role strain, autonomy, and systemic exposure to trauma. Without institutionalizing such thoughtful and safe standards, we risk perpetuating cycles of crisis, moral injury, and preventable collapse.

Importantly, this is not just about individual health workers—it's about systemic resilience. A system that depends on heroism is not resilient—it's brittle. We have seen this brittleness again and again, as clinician shortages lead to canceled surgeries, delayed diagnoses, and increased medical errors. Sustainability in healthcare cannot be decoupled from the sustainability of those who make care possible.

## Conclusion

If we are serious about building resilient, equitable, high-functioning health systems, we must begin by safeguarding the foundation of care: the people who provide it. And that starts not only with policy and practice, but with the kinds of questions we choose to ask—and whom we choose to design for. The systems we build are only as resilient as their weakest link. In healthcare, that link is not technology or policy alone, but the human beings who navigate and deliver care under constant pressure. A healthcare system that fails to prioritize the well-being of the people who provide care is a system on the verge of collapse.

## Contribution statement

The author was solely responsible for all aspects of this paper and has read and approved the manuscript.

## Supplementary Material

qxaf123_Supplementary_Data
